# Bibliographical Notices

**Published:** 1849-03

**Authors:** 


### BIBLIOGRAPHICAL NOTICES.

*The Elements of Materia Medica and Therapeutics.* By JONATHAN PEREIRA, M. D., F. R. S. and L. S., Fellow of the Royal College of Physicians in London; Vice President of the Royal Medical and Chirurgical Society, &c. &c. *Third Edition, enlarged and improved, including notices of most of the medicinal substances in use in the civilized world, and forming an Encyclopædia of Materia Medica.* Vol. 1. 8vo. pp. 897. London: 1849.

A new edition of this excellent work has been announced in the English periodicals for many months; and we have now the first volume. To appreciate the difference between it and its prototype of the former edition, let us see what its distinguished author says in the Preface in regard to the whole work:

"The author has endeavoured to render this work more worthy of the marks of approbation bestowed on former editions. Several portions of it have been entirely re-written, some have been curtailed, others enlarged, and every part has been carefully corrected, and, it is believed, much improved. Numerous recent discoveries in natural history, chemistry, physiology, and practical medicine, relating to the materia medica, have been embodied in this edition, which the author ventures to hope will be found to contain a faithful outline of the present state of pharmacological knowledge. Notices of many of the less frequently employed medicinal substances have also been added, so that the work now embraces an account of the chief medicinal agents used in the civilized world, and may be said to form an *Encyclopædia of Materia Medica*." p. vi.

Of such a designation it is, indeed, eminently worthy; for although not entirely *à portée* with the existing condition of the results derived from experience with all remedial agents everywhere, it is markedly cosmopolitan in its scope; and the author's knowledge of the German enables him to make frequent references, which would have escaped one not similarly endowed.

Dr. Pereira's "Elements"—"System" would have been a more appropriate designation—has been regarded everywhere as a rich mine of valuable information appertaining to the topics on which it treats. Its therapeutical portion is, however, less imposing than its accuracy in every thing regarding the history and properties of the various articles of the materia medica, especially in their pharmacognostic relations. In these respects it is more full perhaps than the excellent dispensatories of Wood and Bache, and of Christison, so well known to, and so highly appreciated by our readers, whilst it necessarily exceeds them in the amount of information connected with general therapeutics. Much additional matter has been added, and not a little on the special application of the particular articles to therapeutics, so that it has been necessary not only to increase the amount of type on each page, but to add materially to the number of pages.

As a work for the library of every medical practitioner and student it appears to us to be essentially demanded;—as one to be taken up and perused continuously by the latter, it is by no means as well adapted. In this respect, however, it but resembles encyclopædiac works in general. It is eminently fitted for the shelf as an authoritative and unerring pharmacological guide: it is not so well fitted for the table of the young student, especially of one who is following the courses of Therapeutics and Materia Medica as taught in the schools. In this relation it has all the virtues and defects of the dispensatories. Our attention has been greatly directed to the treatises on Therapeutics and Materia Medica of various countries; and we have been struck with the differences amongst them in scope and execution,—amongst those that have appeared within the last few years more especially. With the English, the main design appears to have been to furnish exact accounts of the history, preparation, properties, characteristics, composition, physiological effects, and therapeutical uses of the various articles. Generally, however, more attention has been paid to the four first objects than to the two last. The work before us is, however, more equable in all respects; whilst those of Ballard and Garrod, and Royle, give so much preponderance to the former objects, that they are scarcely adapted as text books or accompaniments to lectures on materia medica, certainly in most, if not all, of our medical schools.

The French and German works, on the other hand, give more attention to the therapeutical than to the pharmaceutical relations of medicines. Such is eminently the case with that of MM. Trousseau and Pidoux—the most important that has appeared on the subject, of late years, in France; and the same remark applies, although less forcibly, to the recent productions of Mitscherlich, Oesterlen and Plagge, now before us. Aschenbrenner's small volume, which we have recently received, entitled "*Die neuern Arzneimittel und Arzneibereitungsformen*" *u. s. w.*, Erlangen, 1848, ("New remedies and their forms of preparation," &c ,) quoted by Dr. Pereira, is little more than a skeleton, although containing, in epitome, a large amount of valuable material to one who is editing a dispensatory or collection of "New Remedies."

Taking the American works on the same subject, we find a greater proportionate attention paid to the therapeutical relations of medicine by them than by the English, and hence, most of them—as those of Chapman, Eberle, Dunglison and Harrison (of Cincinnati)—adopt a physiological classification, to which they adhere in the description of individual articles of the materia medica.

As text books, then, to courses of lectures on Therapeutics and Materia Medica, as delivered in most of our colleges, the French,

German and American treatises are better adapted than those of England. We allude especially to treatises of modern production—within the last few years, that is. We have never been of opinion, that encyclopediac works, like the one before us, or dispensatories—no matter how valuable they may be in their legitimate sphere—are good accompaniments as class books. It is rare for us to meet with any youth, prior to his admission into the office of his preceptor, who has much, if any, knowledge of botany or of zoology; and therefore, to the mass, it would be almost as profitable to read or attempt to read Chaldaic as the botanical or zoological portions of the pharmacological works, which are apt to be placed in the hands of the tyro at the very threshold. What better mode of impressing him with distaste for the whole affair than to make either of the great dispensatories, or the encyclopediac work before us, his "first book" of medicine, and require him to study or even to peruse it? He meets with botanical and zoological, not to say chemical descriptions, of which he knows nothing; and if—as we admit—there is an ample amount of intelligible matter, if properly culled for him, in any of these works, who is to make the selection? Certainly not—in many cases—the preceptor, who is too often as uninformed as the student. It has always appeared to us, that works, which give the due amount of information for the practising physician, without attempting to exhaust the subject, are best adapted for the use of one preparing for the practice of his profession; whilst the more elaborate treatises, that regard the articles of the materia medica in their congenerous relations also, are better fitted for one who desires, after his purely medical education has been completed, to prosecute the subject still farther. In all these observations, however, we have relation to the system of instruction as pursued in the different medical schools of this country. Nothing would delight us more than to see a competent knowledge of botany and zoology required of every medical student, as a branch of preliminary or simultaneous study; but it is not probable that so desirable a change in established usages will be speedily effected; nor, indeed, does it seem to us to be very practicable.

In the present edition of his work, Dr. Pereira has "attempted"—to use his own modest expression—a new physiological classification of the articles composing the materia medica—" with what success"—he adds—" the author leaves others to judge. He would remind those who may disapprove of it, of the insuperable" [the epithet is too strong] " difficulties which stand in the way of a satisfactory and unobjectionable classification of medicines on a physiological basis; and he would say to the critic, in the language of Horace,

> 'Si quid novisti rectius istis,
> Candidus imperti, si non, his utere mecum.' "

Deeply impressed with the difficulties to which Dr. Pereira refers, we are disposed to be charitable with every fresh attempt made in the right direction. Dr. Pereira's has the merit of systematically arranging the most important agents, although doubts may exist as to the propriety of positions which he has assigned to individual articles; and still more as to the necessity of many of the subordinate heads, which he has introduced. But we extract it, in order that our readers may judge for themselves:

" Class I. Topical remedies acting mechanically.

- Mechanical antidotes,

- Mechanical purgatives and anthelmintics,

- Dentifrices.

Class II. Topical remedies acting chemically.

- Caustics.

- Astringents.

- Chemical antidotes.

- Disinfectants.

Class III. Topical remedies acting dynamically.

- Acrids.

- Emollients.

Class IV. Hæmatics or remedies acting on the blood.

- *a.* Physically.

  - Diluents.

  - Inspissants.

- *β.* Chemically.

  - Spanæmics or impoverishers of the blood.

    - *a.* Thirst quenching and refrigerant.

    - *β.* Resolvents or liquefacients.

      1. Alkalines.

      2. Salines.

      3. Iodics and bromics.

      4 Sulphurosa

      5. Mercurialia et antimonialia.

    - *γ*· Antispasmodic.

    - *δ*· Plumbeous or saturnine.

  - Hæmatinics or enrichers of the blood.

- *γ*. Dynamically.

Class V. Pneumatics or remedies acting on the organs of respiration.

- Affecting the muscles of respiration.
- Affecting the aërian membrane.
- Diminishing the want of breath.
- Influencing the calorific functions
- Refrigerants.

Class VI. Neurotics or remedies acting on the nervous system.

- 1. Cerebro-spinals

    - 1. Affecting the mind; phrenics.

    - 2. Affecting sensibility; æsthetics

        - *a.* Strengthening it; hyperæsthetics.

        - *β.* Lowering it; anæsthetics.

    - 3. Affecting voluntary and reflex spinal movements; cinetics.

        - *a.* Affecting the tonicity of muscles.

            - *aa.* Augmenting it; tonics.

            - *ββ.* Depressing it; relaxants.

        - *β.* Affecting the irritability of muscles.

            - *aa.* Augmenting it; spastica.

            - *ββ.* Diminishing it; paralytica.

        - *γ.* Affecting volition.

        - *δ.* Affecting the reflex spinal functions.

    - 4. Affecting sleep; hypnics.

        - *a.* Causing it; hypnotics.

        - *β.* Preventing it; agrypnotics,

- 2. Ganglionics.

    - 1. Affecting the heart and arteries.

        - *a.* Exciting them; stimulants.

            - *aa.* Etherec-oily vegetables.

            - *ββ.* Resinous.

            - *γγ.* Ammoniacal and empyreumatic.

            - *δδ.* Animal excretions.

            - *εε.* Phosphorus.

            - *ζζ.* Spirituous and etherial.

        - *β.* Depressing them; sedantia.

    - 2. Affecting the alimentary canal.

Class VIII. Cæliacs or remedies acting on the digestive organs.

- 1. Enterics; anthelmintics.

- 2. Hepatics.

- 3. Splenics.

- 4. Sialics and pancreatics.

- Class VIII. Eccritics, or remedies acting on the excernent system.

    - 1. Augmenting secretion.

        - 1. Errhines.

        - 2. Expectorants.

        - 3. Emetics.

        - 4. Cathartics.

        - 5. Diaphoretics.

        - 6. Sialogogues.

        - 7. Cholagogues.

        - 8. Diuretics.

    - 2. Diminishing secretion.

    - 3. Altering the quality of the secretions; lithics.

- Class IX. Genetics, or remedies acting on the sexual organs.

    - 1. Affecting the organs,

        - aphrodisiacs,

        - anaphrodisiacs.

    - 2. Affecting the uterus,

        - emmenagogues,

        - ecbolics."

It must be admitted, that the divisions and subdivisions of this table are sufficiently ample—perhaps in some respects unnecessarily so; but—as before remarked—we are not disposed to find fault with any arrangement that includes all the various remedial agents, or that finds a place for every one which is admitted to possess decided potency in the cure of disease. Under "*resolvent or liquefacient spanæmics*," the "*liquefacientia*" of Plagge—as we stated in a former number of this journal—and other German pharmacologists, and of the last edition of his own work, Dr. Pereira places alkalines, salines, iodics and bromics, sulphurous agents, mercurials and antimonials. Arsenic is not, however, there, nor do we find other articles that modify nutrition, and were in older periods classed, with numerous others, as "alteratives." Arsenic appears under the suborder *Spanæmica antispasmodica*, along with the sulphate of, and ammoniated, copper, the nitrate of silver, the trisnitrate of bismuth, and the oxide and sulphate of zinc.

It is proper, however, to say that under arsenious acid the author observes: "On the whole, it is impossible, I conceive, in the present state of knowledge, to designate the medicinal effect of arsenic by any term, which shall briefly but characteristically declare its physiological properties. The terms *tonic* and *antispasmodic* are quite insufficient for the purpose; nor am I satisfied with the designation *antispasmodic spanæmic*, before" [in a former part of the volume] "given to it." p. 663.

In this want of satisfaction we participate; but we go farther, and consider the evidence, derived from our knowledge of the *modus operandi* of the different agents classed under the resolvent or liquefacient and the antispasmodic spanæmics, to be insufficient to establish such suborders. All that we seem to know of the remedial agency of the former is, that they modify nutrition—in all probability through some change impressed on the blood—and in this manner remove the different forms of dyscrasy in which they have been found efficacious; but that they do this as "spanæmics" or "impoverishers of the blood," or as "liquefacients" of the morbid formations, appears to us to be wanting in confirmation and even in probability. And in respect to the "antispasmodic spanæmics:" the more the action of reputed "antispasmodics" is investigated, the more must we be satisfied, that we are not possessed of any agent that can be considered as endowed with positive antispasmodic virtues. Spasm is primarily a nervous phenomenon, which may be the result of a sthenic or an asthenic condition; so that remedies possessed of antithetic effects as vital agents, may be entitled, according to the condition of the system, to the epithet "antispasmodic." Assafœtida, castor, dracontium, &c., which are repugnant to taste and smell, and therefore are well calculated to excite a new and powerful impression on the nerves of gustation and olfaction, and are withal excitant to the nerves of the digestive tube, have usually been ranked as "direct antispasmodics;" yet their action is obviously of the most indirect kind; and not a particle of reason, we think, exists for the belief, that any antispasmodic has a direct action on the muscular fibre, by which it can resolve spasm, as has been maintained by some. The phenomena of spasm are produced by erethism in some portion of the nervous system,—when general, of the reflex portion more especially; and accordingly the efficacious remedies are such as act most powerfully on the nervous system. Who, in a case of tetanus or hydrophobia, or of spasm from luxated bone, would dream of reposing his confidence in such "direct antispasmodics" as assafœtida, castor, valerian or skunk cabbage? But we are discussing the order of arrangement adopted by the author, when we had designed to pass it by without any protracted comment.

To the immense labour, the signal accuracy, the vast erudition of its author, not only in pharmacology, but in most departments of his profession, the volume before us bears unquestioned testimony. In every work of the kind having the character of an Encyclopædia or Dictionary of science, it is usually easy to detect omissions; yet in the volume before us these are at least as few as in any similar undertaking. Many of the works on the subject published in Germany, France, England and this country, have been referred to by Dr. Pereira, with suitable acknowledgments; others have evidently not been available to him.

The ectrotic employment of the tincture of iodine externally in small pox we find has escaped him—perhaps on account of the experiments of Dr. Crawford, of Montreal, and of Dr. Samuel Jackson, of this city, (formerly of Northumberland,) and of others not having reached him. They are referred to in the fifth edition of the "New Remedies" of Dr. Dunglison, which he had not received, as he quotes from the fourth edition;—but in a production of such rare excellence, and of such a magnitude, omissions of the kind are scarcely blemishes, and can be readily obviated hereafter; for we trust the work is destined to see many editions.

We dismiss, then, the consideration of the first volume of this "Encyclopædia of Materia Medica," with the confident expectation, that the second volume will prove entitled to the same place in the estimation of the profession as the one before us, and with the hope that we may speedily have an opportunity to herald its advent on this side of the Atlantic.

---

*A Manual of Physiology.* By William Senhouse Kirkes, M. D., *Assisted by* James Paget, Lecturer on General Anatomy and Physiology at St. Bartholomew Hospital. *With one hundred and eighteen illustrations on wood.* Philadelphia: Lea & Blanchard, 1849.

The well known industry of the author and his collaborator, (both of whose names are familiar to us from their connection with the "Reports" on Anatomy and Physiology,) is sufficient warranty for the faithfulness in execution of the work before us. It was originally intended as a compendium of the larger treatise of Müller, and many of its chapters, viz.: those on *Motion*, the *Voice* and *Speech*, the *Senses*, *Generation* and *Development*, are chiefly abstracts of corresponding portions of that work, and the supplement of Dr. Baly and the author. It was found, however, that the advance of physiology had so increased, and modified the facts of the science, that Müller's elements could only be employed as an authority, and the labour of the author in collecting and sifting these facts and opinions has resulted in the production of the volume whose title stands at the head of this article.

In looking through the work, we find but little to carp at, and that little rather in matter of opinion than of facts. In some places the author's style is inverted, and on this account is often obscure.

The first three hundred pages are occupied with the nutritive functions, in the arrangement of which, the author has departed somewhat from the usual plan, without, in our opinion, improving it. The first three chapters are devoted to a brief account of the chemical and structural composition of the human body, and the vital properties of the organs and tissues. We confess, that we would have preferred to see more space occupied with these important subjects, to the exclusion of the minute detail on some other points; organic chemistry, and general anatomy are now too intimately associated with the science of physiology, and are too essential aids to its correct understanding, to be so summarily dismissed. And yet it seems unjust to the author of a work like the present, confessedly a compendium of physiology, to expect the fulness and minuteness of detail that is demanded by the advanced student, since he expressly states that only so much of these kindred sciences is introduced, as will serve to remind the reader of knowledge already acquired.

In the section on *vital properties*, the author enumerates three, viz.: 1st. The *formative force*, or that by which living bodies are able to form themselves out of materials dissimilar from them, as, for example, when the ovule developes itself from the nutriment of the fluids of the parent, or when a plant, or any part of one, grows by appropriating the elements of water, carbonic acid, or ammonia, or when an animal subsists on vegetables, and has its blood and tissues formed from these articles of food. 2d. *Contractility.* —This consists in the power which certain tissues have during life of contracting, or shortening themselves in a peculiar manner, on the application of stimuli. It differs from the simple elasticity, in the fact that it exists only during life, and is developed either by the direct application of stimuli to the tissue itself, or to the nerves ramifying in and upon it; it is inherent in the tissue, however, and is essentially independent of the nerves. 3d. The power of *conducting*, or *transmitting* stimuli, or impressions. This is ascribed to the nerves alone, and, according to the author, constitutes another vital property. It is one which manifestly belongs only to the animal kingdom, and can have no existence in the vegetable organism, which is entirely destitute of nerves. On this account it does not square with the ideas of many physiologists in relation to vital properties, which are defined as properties possessed by every living organism, *vegetable* as well as *animal*.

Chapter IV. contains an able description of the blood, its chemical composition, vital properties, and mode of development. In this chapter, the author alludes to the *vexata quæstio* of the cause of the change of colour in venous and arterial blood, briefly noticing the theories of Liebig, Mülder and Stevens, with a decided leaning, however, to that of Mülder, which attributes the change of colour to a change in the shape of the corpuscles, without any relation whatever to their chemical composition.

In the section on the vital properties of the blood, an abstract of the lectures on the life of the blood, delivered by Mr. Paget, at the College of Surgeons, in May, 1848, is presented to the reader. In this Mr. P. describes two sets of blood corpuscles, as developed at different periods of life, a first set which exists alone in the blood till the chyle and lymph begin to be formed, and a second which is formed from the lymph and chyle corpuscles, and gradually supersedes the first. The corpuscles of the first set, are, in the first instance, part of the embryo cells, which form the mucous or vegetative layer of the embryoes in mammalia and birds, and the whole inner surface of the vitelline membrane, in the embryoes of fish and reptiles. In the latter class, certain of these cells are laid out in the plan of the future heart and blood vessels, before the walls of those organs are yet formed, and before the blood has begun to move. These cells are described by Vogt, Kolliker, and Kramer, as large, colourless, and of a spherical form, filled with yellowish particles of a substance like fatty matter; each cell having a central nucleus, which is much obscured at first by the forementioned fatty particles. In their development into the blood corpuscles, there is a gradual clearing up of the contents, an acquirement of blood colour, and of the elliptical flattened form, with the more prominent appearance of the nucleus. A similar change is doubtless effected in birds, though it is not so readily watched as in the batrachian embryoes, in consequence of their more rapid development. In the mammalia, the earliest blood cells appear also to be a portion of the vegetative or mucous layer of the germinal membrane, and they have the same general appearance as those before mentioned. In these there has been observed a process of multiplication by bi-partition of the nucleus, each half of which, either by appropriating half the cell, or by developing a new one around itself, becomes the central nucleus of a new cell, differing from the parent, from which it escapes, only in being smaller and more generally circular. The subsequent changes resemble those already described; they gradually assume the red colour, and other outward appearances of the red corpuscles. In the development of the embryo, when the lymph and chyle begin to appear, and their corpuscles are added to the blood, they supersede those produced in the manner just described, the old set disappearing as the new set appear, until finally, in an embryo two months old, they would not be found at all, unless in a case of arrested development.

The author then goes on to describe the mode in which, at that time, as well as in after life, the white or colourless corpuscles are converted into the red corpuscles of the blood, thus explaining the mooted question of the origin of the latter. The white corpuscle, which at first is tuberculated, and contains many granules, becomes smoother, paler, less granular, and more dimly shaded. In these stages, the cell wall may be easily raised from its contents by water or acetic acid, and according to the stage of development, so are the various appearances which the cells, thus acted on, present. As the development progresses, the cell wall can no longer be raised from its contents, the corpuscle obtains a red tinge, the contained granules disappear, with the exception of the one, which sometimes remains a longer time, giving rise to appearance which has been mistaken for a nucleus. The blood colour now deepens, the corpuscles become smoother, bi-concave, smaller by condensation, and heavier, and, at the same time, more liable to corrugation and to collect in clusters. This latter is the condition in which they seem to live the longer and more active part of their lives. This mode of production appears to be continued throughout life; new corpuscles never appearing to be produced from the germs of old ones; but when they have past their perfection, they degenerate, and probably liquefy. Herein is a provision for the welfare of the body, for if the blood corpuscles were, like many cells, derived from germs formed in their predecessors, then every loss of blood would involve the loss, not only of the corpuscles escaping at the time, but of all those that in after time would have descended from them. But as new blood is made by the lymph and chyle, its losses can soon be repaired, provided chylosis and lymphosis are not interfered with.

This doctrine is at variance with that of other physiologists to the effect that the corpuscles of the blood are reproduced by a species of fissiparous generation, each individual cell dividing itself into six new cells, according to some, or into two only, according to others, their primary origin in the embryo, being common with that of all the other tissues, from granules or cell germs. It is also irreconcilable with the doctrine of the functions of the white or colourless corpuscles, as set forth by Dr. Carpenter. According to this author there are found in both the vegetable and animal organisms, cells which are developed in a temporary manner; growing and arriving at maturity, and then disappearing apparently without having performed any particular function. This is seen in the albumen of the seed, and in the yolk of the egg. It is also seen, according to Dr. Barry, in the germinal vesicle. This-cell life can scarcely be supposed to be called into existence without some definite end or purpose, which seems here to be—the conversion of the crude alimentary materials into organizable fluids. A number of instances are cited in which this temporary cell-life is displayed, the result of which appears to be the conversion of the aplastic into plastic material.

Both in the blood and in the chyle and lymph cells are found identical with those described before in the origin of the blood corpuscles. In all these fluids the conversion of the aplastic albumen into the plastic fibrine is continually taking place, which conversion is said to be the function of these floating cells, the white corpuscles. In corroboration of this opinion, it is further stated that the colourless corpuscles are always most abundant where fibrine is being elaborated, as in the lacteal vessels in their course from the intestine to the thoracic duct, and likewise in parts where inflammation is present, under which latter circumstances there is an increase both of the fibrinous element, and also of the white corpuscles.

We are free to confess that this doctrine accords more with our preconceived idea of the function of the white corpuscles than that of the work under notice; their larger size, granular appearance, higher refracting power, and greater firmness, seeming irreconcilable with their conversion into the smaller blood disc. But, unfortunately, it leaves us still at sea in regard to the origin of the latter, a fact which rather inclines us to adopt the doctrine of Dr. Kirkes. The chapter concludes with an exposition of the *purposes* of the blood thus developed and maintained. "1st. To provide materials appropriate for the nutrition and maintenance of all the parts of the body. 2d. To convey to the several parts oxygen, whether for the discharge of their function, or for combination with their refuse matters. 3d. To bring from the same parts those refuse matters, and convey them to where they may be discharged.

*Chapter V.* is on the circulation of the blood. It treats of the *Action of the heart;* of the *Action of the valves of the heart;* of the *Sounds and impulse of the heart;* of the *Frequency and force of the heart's action;* cause of the *Rythmic action of the heart; Effect of the heart's action;* of the *Arteries, The pulse; force of blood in* the *arteries; The Capillaries and the veins.*

In the explanation of the sounds of the heart, the author adopts the opinion of Müller, as to their cause, viz.—the contraction of the muscular fibres and the impulse against the walls of the chest, scarcely taking into account the agency afforded in their production by the rush of the blood through the narrow orifices of the aorta and pulmonary artery.

From the experiments of Cruveilhier, it appears that the first sound is heard in its greatest intensity over the very spot in which the second sound is heard most distinctly, viz. at the origin of the pulmonary artery and aorta, and that a distinct purring sensation can also be felt at the same point. Without, therefore, denying the participation of muscular contraction in the first sound, Cruveilhier's observations appear to establish the fact, that the prolonged rush of blood through the orifices of the aorta and pulmonary artery, and the throwing back of the semilunar valves, are the principal causes in its production.

In regard to the second sound, he adopts the usual explanation of its cause, viz. the sudden shutting down of the semilunar valves.

In the section on *The Arteries*, the author establishes their muscularity, but seems to be at fault in relation to its function, not acknowledging that it can assist in the propulsions of the blood, but that it merely regulates the diameter of the vessels in proportion to the amount of the circulating fluid. He thinks that the muscular coat can take no part in the propulsion of the blood, inasmuch as it has no alternately dilating and contracting power, seeming to forget that the stimulus of distention created by the successive jettings of the ventricle is sufficient to excite the contractility of the muscular fibres, and thus assist in the propulsion of the blood.

In the section on *The Circulation in the Capillaries*, the author says, in alluding to the force of the heart: "There is, therefore, no need of an hypothesis of any action of the capillaries for the regular propulsion of the blood through them, nor is it probable they have any such office."

In this point, also, he adopts the opinion of Müller, based upon the observations of Wedermeyer and Dr. Marshall Hall, that "even in the state of greatest debility, the action of the heart is sufficient to impel the blood through the capillary vessels." He admits that "under certain circumstances, the capillaries can exercise an influence on their contents; but that this can be explained by the action of the small arteries, or by the relation which exists between the tissues and the blood.

In order to establish the existence in the capillaries of a motive power, independent of the heart, it is only necessary to cite a few instances in which it is manifestly present. In the embryo the first movement of the blood is seen in the vascular layer of the germinal membrane, before a heart is formed, and it is known that these first movements are *towards* the body of the new being, and not from it, as a centre. The circulation also, is carried in the acardiac fœtus, and the development of the different organs is often as complete as in the perfectly formed fœtus. Dr. Carpenter, whom we again cite, on this point, says that the movements of the blood in the capillaries of cold-blooded animals, after complete excision of the heart has been repeatedly witnessed, and that after most natural deaths the arterial system is found empty; and further, that a real process of secretion not unfrequently occurs after somatic death has taken place. Urine has been poured out by the ureters, sweat by the skin, and other peculiar secretions by their glands; not one of which could have taken place, had there been no capillary circulation.

In a paper that has been received since the commencement of this article, additional evidence in favour of an independent capillary circulation has been received.* In this it is shown that in a vast number of instances the capillary circulation was carried on to such an extent after death, as to cause the blood to jet from a vein as in the operation of venesection. The experiments were, for the most part, performed on yellow fever patients, and generally, immediately after death.

"In the experiments on post-mortem venesection, elevated points and positions were chosen, when practicable, in order to cut off all possibility that the result might be influenced by gravity; at the same time, care was generally taken that the orifices in the arm, forehead, external jugular, &c., should be elevated but *little* above the level of the highest part of the body, for fear that gravity might favor the result—which it always does to a very limited extent at the first moment, though it makes the subsequent part of the experiment far more striking and brilliant. Sometimes, the elevation was taken, by pouring water on a plank, the latter resting on the body. All the blood, at the moment, in the *distal* end of the vein, that is, beyond the orifice, would, if raised above the level, be discharged from gravity; perhaps an ounce or more would, in some cases, thus be lost in emptying the vein, but then, *no more could get there*, to replace the *first*, unless by a power altogether *different from gravity*. Hence, it appears, that all the blood, with this inconsiderable exception, after the *first moment* flowed by capillary forces—without the aid of, or rather *against*, all that is called mechanical principles—against all the chemical forces incidental to respiration—against ganglionic forces, for the ganglions were often cut away,—and, always, without aid from the heart.

*Case.*—1841. Yellow Fever. A man aged 34, died—was brought immediately, &c.; a vein in the left arm was opened—the blood flowed

* Researches Critical and Experimental on the Capillary Circulation. By Bennet Dowler, M. D., of New Orleans.

freely—on moving the muscles it jetted, and upon ligation formed an arch of about eighteen inches in diameter, as in ordinary venesection. The left jugular being opened in the usual manner, bled copiously without jetting. The abdomen, and chest, were opened without delay; one of eight or ten little twigs of nearly equal size, belonging to the coronary vein of the heart, was opened upon its highest point; the blood shot out in a small, strong stream, a pint being discharged in a few minutes. The omenta, and mesentery, were beautifully and forcibly distended, especially, in the venous vessels. The cavas discharged from three to four pounds of blood.

*Case.*—1841. Yellow Fever. A man, aged 25, died, lying on his right side—soon after he was placed on his back—in which position, in fifteen minutes after death, he was opened. The liver, which was very brittle, was penetrated upon its highest, convex surface; in a few minutes, three and a half pounds of blood were discharged. The blood was taken up from the abdomen, in a sponge, and squeezed out into a vessel and measured. A puncture with the *finger*, would not be likely to divide any large vein, nor even in the living state, produce much hæmorrhage. The capillaries of the portal system, supplied nearly all this blood, so rapidly delivered from the abdominal viscera. (At the close of life, this man had nasal hæmorrhage.) Those who practice anatomical injections, know that only a few ounces of wax can be forced into the vessels of the liver."

In the author's own language, "These facts are not referable in their main features to any physical or non-vital law; they are the last acts of vitality, before yielding complete and endless submission to the laws which govern the inorganic world—the preludes to the entire extinction of life." The cases quoted are but two out of a number of similar ones; the subjects dying of yellow fever, apoplexy and cholera; and it is difficult to explain them on any other ground than that of a post mortem capillary power independent of the heart's action.

At the first blush it seems as though they were capable of explanation on the ground of pressure upon the superficial veins during the post mortem rigidity, just as muscular contraction will facilitate the flow of blood in venesection, but this will not explain the post mortem circulation in the mesentery, the venæ cavæ, the liver, and the brain. The upholders of the capillary power owe a debt of gratitude to Dr. Dowler for the strong ground that his valuable contribution to Physiology enables them to assume, and we would strongly recommend his essay to the careful perusal of the Physiological student as well as to the skeptical on this subject. In addition to the establishment of the points under consideration, the experiments of Dr. Dowler also confirm the doctrine of a propelling power in the chylous vessels, as is seen in the following cases in which the external jugular was opened and the chyle made its appearance in the escaping blood. It will be seen that in this instance the blood moved in a retrograde manner. This is explained, however, by Dr. Dowler, as follows:

"The capillaries filled the veins rapidly. The forces in the cavas, contended face to face. The right side of the heart had no outlet. The equilibrium between the ascending and descending cavas was broken. The latter was weakened by the force or pressure turned off by the orifice, in the jugular. The pressure from below caused a retrograde movement—a very short one—towards the point where there was the least pressure, that is, the orifice in the vein.

In the early part of the experiment, particularly after the jetting of blood was changed to a stream down the neck, I observed, for a long time, that is while the blood flowed, what then appeared to me an inexplicable phenomenon, namely, white, milky streaks, or soft flocculent masses, passing out of the orifice in the blood, or rather swimming on the top of the current, a diffused drop or white flocculent cloud of this kind, was carried out once or twice every second. The subsequent dissection fully explained this. The stomach contained nothing but water, of a sourish scent, with a few, very slender, tendinous fibres, apparently of beef, perhaps not amounting to five grains; but the quantity of chyle, of a pale milky, white color, thinner than paste, in the small intestines, was very extraordinary. The lacteals were gorged with this liquid,—the very same kind that I had seen passing from the jugular. I had, fortunately, opened the left jugular, near the place where the chyle is poured into the subclavian, so that it found an easy exit, along with the refluent current of blood, which latter, as before suggested, passed, or a part of it, in a retrograde course, proving at once, the independent forces of the lacteal, and blood vessels,—modified in this instance, however, by an important law of hydrodynamics, which changed their physiological directions.

This last case, (not to name others), fruitful in physiological suggestions, shows that although life, in its popular or utilitarian sense, be completely extinct, several important sub-lives, in tissues, and several functions may survive for a considerable period, as manifested in the heat generating process, in muscular contractility, in capillary action, and in the chylous circulation."

These facts, independently of the arguments of Mr. Daniell, Dr.

Draper, Dr. Carpenter, and others, are to our minds capable of fully establishing the points under consideration, to wit, the existence of a capillary power independent of the heart's action, and vital in its character.

Our waning space warns us that it is time to hasten to the consideration of the remainder of the work under notice, which we do with the observation that the remainder of the chapter on the circulation seems fully posted up to the existing state of the science.

In the chapter on Respiration, a full analysis is given of the valuable paper of Mr. Hutchinson upon the *vital capacity* of the chest, and the influence which age, sex, and size exert upon it. This vital capacity is indicated by the amount of air that is capable of being expelled from the chest by the greatest possible expiration, after the deepest possible inspiration. It will readily be seen that if any certain average can be established as regards the vital capacity, then any falling short of this would be an indication of disease, even before its revelation by physical signs, as was seen in the case of Freeman, the American giant, referred to by Mr. H.

Chapter VI. is on Animal Heat, a title, by the way, which seems to us objectionable in a Physiological treatise, since it conveys the idea that the production of heat is a function exclusively animal, and not existing in the vegetable. This impression is scarcely corrected by the slight reference to the evolution of heat in vegetables on page 182. The old title of *Calorification* seems preferable.

Chapter VIII. and IX. treat very fully and ably of the subjects of Digestion and Absorption. In chapter X. on Nutrition and Growth, we have been anticipated in a criticism which we had marked, by a trans-atlantic reviewer.* On page 23, it is laid down that a certain influence of the nervous system is essential to healthy nutrition.

We are ready to admit that the function of nutrition may be *modified* by the agency of the nervous system, but we cannot agree that the influence of the nervous system is *essential*, simply for the reason that the function of nutrition and growth is carried on in the ovum at a period anterior to the development of a nervous

* British and Foreign Medico-Chirurgical Review, Jan., 1849.

system, and that it is also manifestly present in the vegetable kingdom, in which nothing analogous to a nervous system can be found; these latter facts, however, are stated by the author also. In the language of the reviewer mentioned above, our own ideas on this subject are expressed. "What is meant," says he, "by the expression a *condition essential*, but that without this influence healthy nutrition is impossible? Nutrition is a property inherent in each tissue, though it may, especially in the higher animals, be influenced by causes acting through the nervous system. It is absolutely necessary to separate those conditions which are essential, from those which are accidental or accessory, if we wish to avoid falling into serious errors."

The last of the functions treated of in this part of the work is *Secretion*, after which *the Nervous System* and *Generation*, and *Development* are considered in detail.

In animadverting upon this part of the work, we cannot omit to notice what is evidently an inadvertence on the part of the author in relation to the laws of optics. On page 406 in speaking of the *cornea*, he says:

"It is in a two-fold manner capable of refracting and causing convergence of the rays of light that fall upon and traverse it. It thus affects them, first, by its density; for it is a law in optics that when rays of light pass from a rarer into a denser medium, if they impinge upon the surface in a direction removed from the perpendicular, they are bent out of their former direction towards that of a line perpendicular to the surface of the denser medium; and, secondly, by its convexity—*for it is another law in optics that rays of light impinging upon a convex transparent surface are refracted towards the centre, those being most refracted which are farthest from the centre of the convex surface.*"

This second law in optics is new to us, and entirely unnecessary, the first being amply sufficient to explain the convergence of rays of light falling upon a double convex lens, as well as the divergence of those that are transmitted by a double concave one, provided the remainder of the law be expressed, that a ray of light passing *from a denser into a rarer medium*, is refracted *from* a perpendicular drawn to the surface of the medium at the point of emergence.

But we have already too much extended this notice, and we are therefore obliged to take leave of this work, which, notwithstanding our criticisms, we believe to be an honest and faithful digest of physiological facts and opinions, fully representing the present state of the science.

In the American reprint, the publishers have improved greatly upon the English manual, by introducing well executed copies of the engravings in the front part of the work, through the body of the book, thus saving the student the annoyance of turning backwards and forwards in referring to them.

As an introduction to the study of the larger works, or as a reference for those who desire to "brush up" their knowledge, we most cordially recommend the manual of Kirkes and Paget to both practitioner and student, with the firm conviction that they will not be disappointed in the end they desire to attain.

---

*An Introduction to Practical Chemistry, including Analysis.* By JOHN E. BOWMAN, Demonstrator of Chemistry in King's College, London. Philadelphia, Lea & Blanchard, 1849 : pp. 302.

The little work, of which the above is the title, has just made its appearance in an American dress, through the enterprise of Messrs. Lea & Blanchard, who have already done so much for this branch of scientific literature. The chief merit of the work in question, is the presentation of an abstruse subject in a manner well suited to the ready comprehension of beginners, whilst the manner of proceeding with reference to analysis in many of the cases instanced, will serve to refresh the memories of the more advanced. The importance of analytical knowledge to the physician is great—he is almost entirely dependent on others for assistance in those cases which are recurring from time to time in his practice. When it is necessary to know the composition of a calculus, of urine, or some other secretion or excretion of his patients, and where his location does not admit of appeal to others, a want of this knowledge deprives him of a powerful aid in his diagnostic examinations.

It is presumed that every practitioner has a certain respectable amount of chemical knowledge acquired in the course of his education. With a set of re-agents, a little simple apparatus, and the book under consideration in his possession, a little attentive and persevering application will place him in a position independent of others in a variety of the cases alluded to; and though, with these few accessaries, he may never hope to be a Liebig, or a Mülder, they will be sufficient to give a value to his opinions in the judgment of others, and certainly in his practice, altogether satisfactory to himself, and beneficial to his patients.

The first part of the work is devoted to Manipulation—the manner of proceeding in the generation of gases—distillation—glass working, blow-pipe operations, specific gravities, the applications of heat, &c. The second part, to the action of re-agents on bases and acids. The third part, to the qualitative examination of unknown substances. The fourth part to quantitative analysis, with a preliminary chapter on the mechanical operations required in its pursuit. The fifth part includes the mode of proceeding in examining urinary and biliary calculi, &c., the manner of assuring one-self of the purity of re-agents.

An appendix to the work embraces many valuable tables of weight, measures, solubilities, specific gravities, &c., and adds much to its value as a book of reference. In conclusion, we would strongly recommend "Bowman" to the attention of our medical friends and readers who have chemical predilections, as well as to those who stand in need of this kind of knowledge, as a valuable contribution to the list of treatises on analytical chemistry.

